# Quantification of airborne SARS-CoV-2 genomic particles in different hospital settings

**DOI:** 10.1038/s41598-021-00761-1

**Published:** 2021-10-28

**Authors:** Luís Fernando Amato-Lourenço, Natália de Souza Xavier Costa, Kátia Cristina Dantas, Suzette Cleuza Ferreira Spina Lombardi, Alfredo Mendroni Júnior, José Angelo Lauletta Lindoso, Felipe Gallego Lima, Regiani Carvalho-Oliveira, Thais Mauad

**Affiliations:** 1 LIM-05, Department of Pathology, Faculty of Medicine, University of São Paulo, São Paulo, Brazil; 2 Institute of Advanced Studies (IEA) Global Cities Program, University of São Paulo, São Paulo, Brazil; 3 Division of Research & Transfusion Medicine, Pro-Blood Foundation/Blood Center of São Paulo, Sao Paulo, Brazil; 4 Laboratory of Medical Investigation in Pathogenesis and Targeted Therapy in OncoImmuno-Hematology (LIM-31), Department of Hematology, Hospital das Clínicas - HCFMUSP, Faculty of Medicine, University of São Paulo, São Paulo, Brazil; 5 Departamento de Moléstias Infecciosas e Parasitárias - Faculty of Medicine, University of São Paulo, São Paulo, Brazil; 6 Institute of Infectious Diseases Infectology Emilio Ribas, Sao Paulo, Brazil; 7 Heart Institute (InCor), School of Medicine at Sao Paulo University, Sao Paulo, Brazil; 8 Faculty of Medicine, University of São Paulo, Dr. Arnaldo Avenue, 455, Room 1150, Cerqueira Cesar, São Paulo, 01246903 Brazil

**Keywords:** Environmental sciences, Diseases

## Abstract

We quantified the presence of SARS-CoV-2 RNA in the air of different hospital settings and the autopsy room of the largest medical centre in Sao Paulo, Brazil. Real-time reverse-transcription PCR was used to determine the presence of the envelope protein of SARS-CoV-2 and the nucleocapsid protein genes. The E-gene was detected in 5 out of 6 samples at the ICU-COVID-19 ward and in 5 out of 7 samples at the ward-COVID-19. Similarly, in the non-dedicated facilities, the E-gene was detected in 5 out of 6 samples collected in the ICU and 4 out of 7 samples in the ward. In the necropsy room, 6 out of 7 samples were positive for the E-gene. When both wards were compared, the non-COVID ward presented a significantly higher concentration of the E-gene than in the COVID-19 ward (p = 0.003). There was no significant difference in E-gene concentration between the ICU-COVID-19 and the ICU (p = 0.548). Likewise, there was no significant difference among E-gene concentrations found in the autopsy room versus the ICUs and wards (dedicated or not) (p = 0.245). Our results show the widespread presence of aerosol contamination in different hospital units.

## Introduction

Severe acute respiratory syndrome coronavirus 2 (SARS-CoV-2), caused by a novel beta coronavirus, was first reported in December 2019 in Wuhan, China, and rapidly spread on a global scale^1^. By April 2021, coronavirus disease had caused over 135 million cases and almost 3 million deaths worldwide^2^. In Brazil, the disease has resulted in major social/economic burdens due to the uncontrolled transmissibility rates that can be attributed to inefficient sanitary protocols and federal negationism. With the emergence of the novel P1 variant with higher transmissibility, Brazil is currently one of the global epicentres of the disease^3^.

Airborne transmission of SARS-CoV-2 has been recognized and demonstrated as one of the modes of viral transmission^2,4,5^. Liu et al.^4^ reported the presence of SARS-CoV-2 RNA in aerosols of different environments in two Wuhan hospitals dedicated exclusively to patients infected with SARS-CoV-2. Similarly, Santarpia et al.^6^ found SARS-CoV-2 RNA in the indoor air of the University of Nebraska Medical Centre in areas occupied by patients with mild and moderate infections. However, to our knowledge, few studies have compared the presence of SARS-CoV-2 genes in the indoor air of hospital wards and intensive care units dedicated to COVID-19 patients versus non-COVID-19 units^7,8^. Since the virus can remain viable and infectious in aerosols for hours^9^, there are serious concerns about the viral contamination of the air surrounding patients and health care professionals, especially in the patients and health care professionals allocated to the non-dedicated sectors.

Due to the high demand for hospital space for infectious patients requiring clinical care, many hospitals in Brazil had to rapidly adapt to create wards and ICUs dedicated to COVID-19 patients. The Hospital das Clínicas of the Sao Paulo University Medical School, the largest tertiary care centre of Latin America, became the reference centre for the more severe cases of COVID-19 in São Paulo^10^. One of the hospitals of the complex created an ICU and another ward exclusively dedicated to receiving COVID-19 patients. In addition, a dedicated autopsy room for minimally invasive autopsies (MIAs) was created in the morgue^11^ because conventional autopsies were forbidden by law in Brazil since March 2020 (the autopsy rooms in the country did not comply with the appropriate biosafety recommendations, especially in relation to the presence of Airborne Infection Isolation Rooms or negative pressure systems^12^).

This issue raises questions such as: what are the different levels of contamination by viral aerosol from SARS-CoV-2 considering different hospital settings? We hypothesized that the concentration of SARS-CoV-2 genomic particles would be higher in dedicated units. Therefore, we quantified the presence of SARS-CoV-2 RNA in the air of different hospital areas (isolation/non-isolation sectors for COVID-19 patients) and in the autopsy room in one of the hospitals within the largest medical centre in Sao Paulo, the epicentre of COVID-19 cases in Brazil.

## Results

Descriptive results of SARS-CoV-2 E- and N-gene concentrations (genomic units/m^3^) from aerosol collections at hospital facilities and the necropsy room, the number of patients, temperature, and humidity are summarized in Tables 1, 2 and 3.

**Table 1:** Descriptive data of the ICUs.

Location	Date	Temperature (°C)	Humidity (%)	Total patients	E-gene (0-non-detected/1-detected)	E-gene quantification (genomic units/m^3^)	N-gene (0-non-detected/1-detected)	N-gene quantification (genomic units/m^3^)
ICU	09/17/2020	20.0	52	18	1	54,422	1	44.78
ICU-COVID-19	09/17/2020	19.8	50	8	1	67,749	0	–
ICU	09/18/2020	20.0	50	17	0	–	0	–
ICU-COVID-19	09/18/2020	20.0	51	8	0	–	0	–
ICU	09/21/2020	20.5	50	18	1	16,446	0	–
ICU-COVID-19	09/21/2020	20.2	50	8	1	5258	1	128.34
ICU	09/22/2020	20.0	51	18	1	158,663	0	–
ICU-COVID-19	09/22/2020	19.8	55	7	1	33,744	0	–
ICU	09/23/2020	21.2	52	17	1	1294	0	–
ICU-COVID-19	09/23/2021	20.0	50	7	1	262,508	1	718.83
ICU	09/24/2020	20.0	50	16	1	29,357	0	–
ICU-COVID-19	09/24/2020	20.2	50	6	1	51,622	0	–

**Table 2:** Descriptive data of the wards.

Location	Date	Temperature (°C)	Humidity (%)	Total patients	E-gene (0-non-detected/1-detected)	E-gene quantification (genomic units/m^3^)	N-gene (0-non-detected/1-detected)	N-gene quantification (genomic units/m^3^)
Ward	09/25/2020	20	52	12	0	–	0	–
Ward-COVID-19	09/25/2020	20	53	9	1	27,754	0	–
Ward	09/26/2020	21.5	50	12	0	–	0	–
Ward-COVID-19	09/26/2020	20	51	8	1	32,341	0	–
Ward	28/09/2020	20.5	51	13	1	49,368	0	–
Ward-COVID-19	28/09/2020	19.9	53	10	1	44,370	0	–
Ward	09/29/2020	20.1	52	13	1	69,699	0	–
Ward-COVID-19	09/29/2020	20	55	11	0	–	0	–
Ward	09/30/2020	20	51	15	1	78,700	0	–
Ward-COVID-19	09/30/2020	20.1	57	13	1	13,925	1	12.45
Ward	10/01/2020	20.5	50	16	1	58,427	1	78.08
Ward-COVID-19	10/01/2020	20.3	56	17	0	–	0	–
Ward	10/02/2020	21	51.5	11	0	–	0	–
Ward-COVID-19	10/02/2020	20.9	54	16	1	26,141	0	–

**Table 3:** Descriptive data of the necropsy room.

Location	Date	Temperature (°C)	Humidity (%)	Total patients	E-gene (0-non-detected/1-detected)	E-gene quantification (genomic units/m^3^)	N-gene (0-non-detected/1-detected)
Autopsy room	10/14/2020	17.9	76	1	0	–	0
	10/16/2020	17.7	75	0	0	–	0
	10/19/2020	17.8	78	0	1	31,756	0
	10/20/2020	17.9	81	2	1	97,605	0
	10/21/2020	18.0	85	1	1	32,717	0
	10/22/2020	17.3	76	1	1	15,575	0
	10/23/2020	17.1	74	0	1	25,414	0

The E-gene was detected in 5 out of 6 samples at the ICU-COVID-19 and in 5 out of 7 samples at the ward-COVID-19. Similarly, in the non-dedicated facilities, the E-gene was detected in 5 out of 6 samples collected in the ICU and 4 out of 7 samples in the ward. In the necropsy room, 6 out of 7 samples were positive for the E-gene. The N-gene was detected in 2 samples at the ICU-COVID-19 and 1 sample at the ward-COVID-19; however, in the non-dedicated facilities, it was detected in 1 sample in the ICU and 1 in the ward. No sample was positive for the N-gene in the necropsy room.

**Figure 1:**
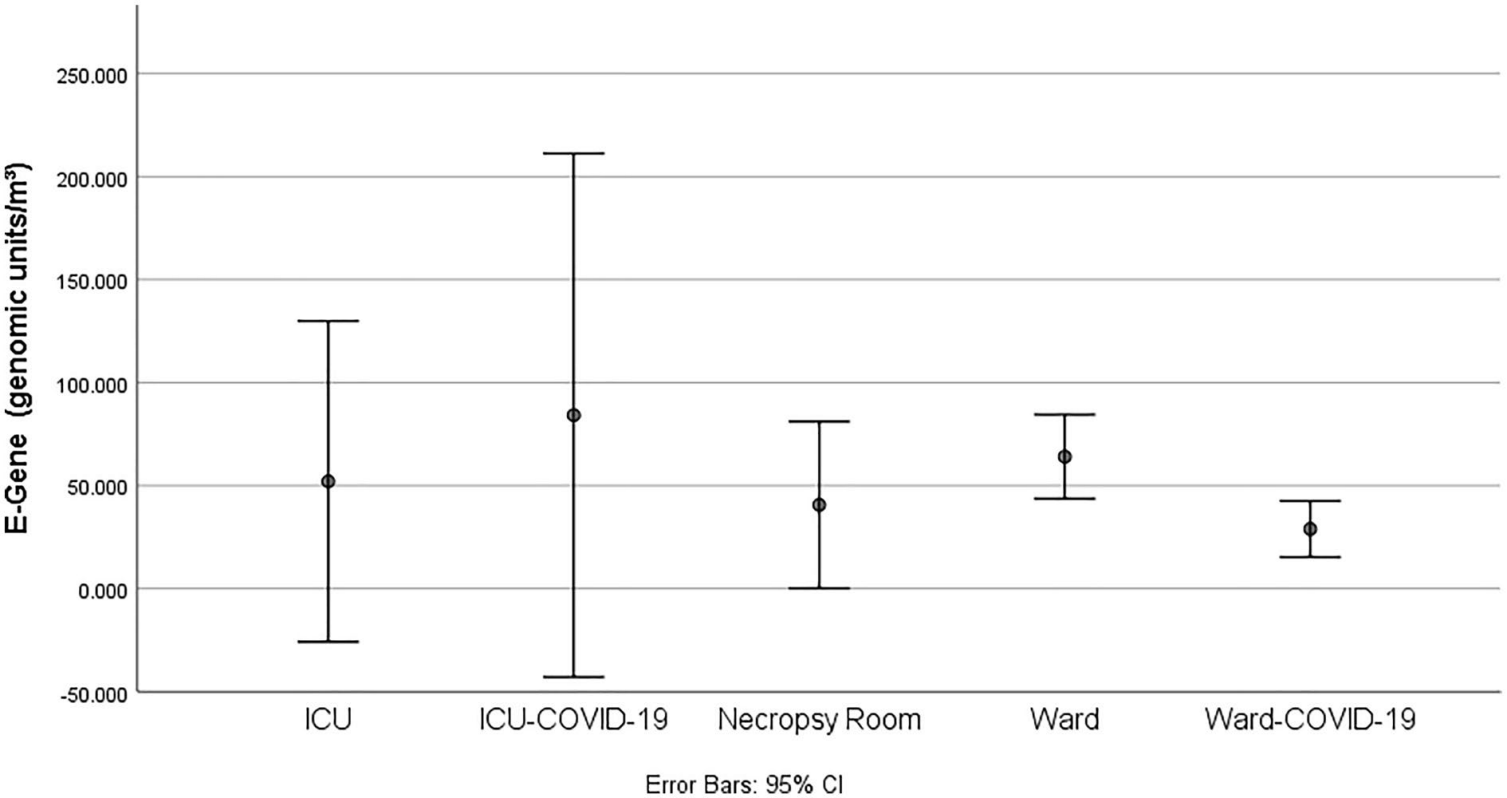
E-gene concentrations according to the sites.

When both wards were compared, the non-COVID wards had a significantly higher concentration of the E-gene than the COVID-19 ward (non-COVID ward median: 64.06 (49.37 to 78.70) genomic units/m^3^; COVID ward median: 27.75 (13.92 to 44.37) genomic units/m^3^, (p = 0.003)). There was no significant difference among E-gene between the ICU-COVID-19 and ICU (ICU-COVID-19 median: 51.62 (5.53 to 262.50) genomic units/m^3^ and ICU median 29.35 (1.29 to 158.66) genomic units/m^3^, (p = 0.548)). Likewise, there were no significant differences (p = 0.245) among E-gene found in the autopsy room (median 31.75 (15.57 to 97.60) genomic units/m^3^) versus the ICUs and wards (dedicated or not) as shown in Fig. 1.

## Discussion

In this study, we compared the presence and concentration of particles containing genomic signatures of SARS-CoV-2 in dedicated and non-dedicated COVID-19 ICUs and wards in a large tertiary hospital; there were no differences between the ICU units, but there was a larger number of E-gene in the non-COVID ward. In addition, in this study, we show the presence of SARS-CoV-2 gene particles in an autopsy room dedicated to MIAs inside the morgue. Our data show a widespread presence of particles containing the SARS-CoV-2 E gene in several areas of our hospital. To our knowledge, this is the first study to investigate the presence of viral particles in an autopsy room.

We detected the SARS-CoV-2 E-gene in 72.7% of all samples and N-gene in 18.2%, with concentrations of both ranging from 1.29 to 262.50 genomic units/m^3^. Stern et al.^8^ previously compared the presence of genomic particles in air samples at three particle sizes (> 10.0 µm, 10.0–2.5 µm, and ≤ 2.5 µm) in the ICU, in the emergency department, and COVID-19 and non-COVID-19 wards; they demonstrated the presence of viral copies in 9% of the samples but had a much lower concentration of units (5–51 copies/m^3^). Our data presented here, regarding the number of positive samples and concentrations, are in contrast to their results. In addition to methodological variations in the studies, the absence of negative pressure in our hospitals could likely explain our higher numbers.

Interestingly, the previous study by Stern et al.^8^ found that the number of positive samples and the concentration of viral particles were higher in the non-COVID-19 wards than in the COVID-19 wards, which agrees with our study here. The higher number of circulating people, especially around the nurse’s station, is certainly a plausible explanation for these increased numbers, despite the same air circulation system within the hospital.

Other studies have detected a much lower number of positive samples in hospital settings compared to our study. For instance, in Hong Kong, where preparedness measures were taken to control nosocomial infections, one in 46 airborne samples was positive for the virus, indicating the effectiveness of preventive measures^13^.

In this study, we collected air samples of sizes < 2.5 µm, which are the particle sizes that are the most likely to deeply penetrate the lungs. Stern et al.^8^ detected the presence of viral copies regardless of the PM collector size. It is possible that our concentrations could be higher if we had measured the total suspended particles.

A limitation of this study is that we did not assess viral viability or infectivity since our filter material inactivates the virus rapidly (Pan et al.^14^). Santarpia et al.^6^ observed viral replication in cell culture for some of the airborne samples collected in hospital wards, suggesting the potentially infectious nature of the recovered virus.

In this study, we found that the concentration of the nucleocapsid gene was lower than that of the E-gene. Passos et al.^7^ also found few positives when testing the samples for only the nucleocapsid gene. Setti et al.^15^ previously obtained more positive samples for the SARS-CoV-2 envelope RNA than for other regions tested. This difference may be related to the integrity of the collected samples.

In Brazil, autopsies were forbidden by law starting in March 2020 due to the lack of adequate biosafety protocols in the rooms that do not have adequate ventilation systems. Our group has been performing MIAs for COVID-19 patients since March 2020 with sealed bodies and personal protection measures as previously described by Duarte‐Neto et al.^11^. The MIA procedure is expected to generate no aerosols. Nevertheless, we were able to detect genomic particles in more than 6 out of 7 samples. Interestingly, none of the autopsy staff tested positive for SARS-CoV-2 or were diagnosed with COVID-19. Similarly, Rakislova et al.^16^ recently published their MIA protocol, also performed in a room without a negative pressure system. None of the staff tested positively or acquired COVID-19, showing the importance and efficacy of adequate personal protection equipment.

In summary, our results show the widespread presence of contaminated aerosols in different hospital units dedicated to or not dedicated to COVID-19 and in the autopsy room, all without negative pressure ventilation systems. Considering the potential transmissibility through aerosols, in a setting without appropriate ventilatory systems and a high number of cases, a situation common to many low-medium income countries, our data support the appropriate use of adequate individual protection and restricted circulation of people in all hospital areas.

## Methods

### Hospital and morgue facilities

This study was carried out in a public university hospital with high medical standards specializing in cardiology and pneumology. Due to the high demand for patients infected with SARS-CoV-2 during the pandemic, the hospital allocated both COVID-19 facilities and non-COVID-19 areas, including intensive care units (ICUs) and wards. During this study, the ICU-COVID-19 and ward-COVID-19 both had individual rooms with independent air conditioning systems but without negative pressure systems. The COVID-19 ICU had a total of 12 beds and the non-COVID-19 ICU 18 beds. The COVID-19 ward had a total of 18 beds and the non-COVID-19 ward had 16 beds.

Complementarily, we conducted sampling in an autopsy room dedicated to MIAs located in the São Paulo Death Verification Service (Serviço de Verificação de Óbitos da capital—SVOC) of São Paulo University.

Hospital policy during the sampling period included restricted visitation, universal masking for staff and patients outside their rooms, elevators with exclusive access to COVID-19 areas, restriction on the number of people on elevators (4–6 people), and mandatory PCR tests for all hospitalizations. At the time of the sampling for this study, COVID-19 vaccines were not yet available in Brazil.

The autopsy room dedicated to MIAs in the morgue had no ventilation system. The room measures 19.5 m^2^ and MIAs procedures were performed by two or three people. During the MIAs, no generation of aerosols was expected. More details on the MIA procedures and individual safety measures can be found in Duarte‐Neto et al.^11^.

### Aerosol sampling

Aerosol sampling was carried out from September to October 2020 in the period between the first and second waves of COVID-19 in São Paulo Municipality (average of 1036 daily cases) (SEADE^17^). However, in this city, there was never a sharp decrease in cases as seen in other countries. The samples were collected for 8 h daily using a MiniVol^®^ sampler (Air Metrics, Innovative Air Sampling Equipment, Springfield, Oregon, USA) containing polycarbonate filters of 47-mm diameter and 0.4-μm pores (Millipore® Burlington, Massachusetts, USA). The MiniVol^®^ was calibrated with a flow of 5 L/min collecting PM_2.5_ fraction. The samplings were carried out at a height of 1.25 m, corresponding to the breathing height of an adult person (Sharma and Kumar^18^). The equipment was cleaned with 70% alcohol between each sampling.

### Details of sampling location

#### COVID-19 ward

The equipment was allocated in the corridor of COVID-19-positive patients, one metre from one of the patients’ rooms and approximately 3 m from the nurses’ station. In front of each room, there was a small station for changing gloves. During the study period, there was one patient in each room. There was no negative pressure system in place.

#### Non-COVID-19 ward

The equipment was positioned in the main access corridor to the ward, adjacent to the nursing station and approximately 2 m from the patients’ rooms.

#### COVID-19-ICU

The equipment was allocated next to the staff entrance to the ICU, adjacent to a workstation and approximately 2 m from the nurses’ station. During the study period, there was one patient in each room. Patients were monitored individually by video camera to avoid unnecessary entry into the rooms. There was no negative pressure system in place.

#### Non-COVID-19-ICU

The equipment was positioned in the main access corridor to the ICU, adjacent to the nursing station and approximately 6 m from the patients’ rooms. This nurse’s station was in an open area with a large circulation of staff.

#### Autopsy room

The equipment was placed inside the dedicated room for minimally invasive autopsies. This room is located on the underground floor of the morgue and has small dimensions (19.5 m^2^) without exhaust systems. The access door to the room remained closed while the procedures were not being carried out.

Samplings for both the COVID-19 and non-COVID-19 sectors were carried out simultaneously. A total of 6 samplings were conducted in dedicated COVID-19 facilities, while 7 samplings were carried out in the non-dedicated sectors and the autopsy room. Field blanks were used and processed simultaneously with the samples. Temperature and relative humidity were measured using a conventional digital thermohygrometer (AKSO AK-28®, ± 1 °C, ± 5% RH).

After the sampling, the filters were collected on-site, packaged in pre-sterilized sealed plastic bags, and immediately stored at − 20 °C. All the materials used in the handling of the filters (tweezers, Petri dishes, among others) were autoclaved before sampling and opened at the time of collection.

### SARS-CoV-2 quantification

#### RNA extraction

Nucleic acids were extracted from the polycarbonate filters. Briefly, the filters were incubated for 3 h at 56 °C in AL buffer (Qiagen, Hilden, Germany) and 100 mg/mL proteinase K (Qiagen, Hilden, Germany). Then, RNA was extracted using a Magna Pure Compact Nucleic Acid Isolation kit (Magna Pure Compact, Roche Diagnostics GmbH, Germany) according to the manufacturer’s instructions.

### Real-time reverse-transcription PCR

SARS-CoV-2 RNA was quantified by an in-house real-time PCR assay that amplified part of the envelope protein (E)^19^ and nucleocapsid protein (N)^20^ genes. Positive and negative controls were included in all amplification reactions. As a positive control, synthetic RNA from SARS-CoV-2 Standard (Exact Diagnostics SARS-CoV-2 Standard, Cat Number #COV019) and RNA extracted from inactivated SARS-CoV-2 were provided by Dr. E. Durigon and Dr. D. Durigon of the University of Sao Paulo, Brazil. Negative controls consisted of the above reaction with all the reagents and eluents without sample.

Real-time PCR was performed using the StepOne System equipment (Applied Biosystems, Foster City, CA, USA) using the methods described previously in Corman et al.^19^. Primer and probe sequences are presented in the Supplemental Material—Table S1. Standard curves were generated from serial dilutions (1:10) of SARS-CoV-2 RNA (Supplemental Material Figs. S2, S3) and converted to genomic units per m^3^. Samples were considered positive if amplification of target regions had a cycle threshold value (Ct) less than 40.

### Statistical analyses

Descriptive data are presented as the median or the mean, depending on the data distribution. The Mann–Whitney U test was used to test for differences in the concentrations of envelope protein and nucleocapsid genes in the dedicated and non-dedicated ICUs. Analysis of variance (one-way analysis of variance (ANOVA)) was used to test for differences in the concentrations of the envelope protein and nucleocapsid genes in the dedicated and non-dedicated wards. The differences in the concentrations of envelope genes and nucleocapsid genes in all hospital facilities and necropsy room were tested by the Kruskal–Wallis test. Statistical analyses were performed using IBM® SPSS® Statistics software (version 26 IBM Corp., Chicago, IL, USA). A value of p < 0.05 was considered statistically significant.

## Supplementary Information

Supplementary Information.

## Data Availability

For primer sequences and standard curve data, see the Supplemental Material. All other data or materials can be obtained from the corresponding author upon request.
