# Supplementary material for: Quantification of airborne SARS-CoV-2 genomic particles in different hospital settings
Source: Sci Rep. 2021 Oct 28;11:21284. doi: 10.1038/s41598-021-00761-1 (PMC8553841; doi:10.1038/s41598-021-00761-1)
Supplement: Supplementary file 1 — Supplementary Information. [file 41598_2021_761_MOESM1_ESM.docx]

**SUPPLEMENTAL MATERIAL**

Table S1. Primer sequences:

| **Target** | **Primer/Probe sequence** | |
| --- | --- | --- |
| SARS-CoV-2 envelope protein gene (E) | Forward | 5’-ACAGGTACGTTAATAGTTAATAGCGT-3’ |
|  | Probe | 5’-FAM-ACACTAGCCATCCTTACTGCGCTTCG- BBQ-3’ |
|  | Reverse | 5’-ATATTGCAGCAGTACGCACACA-3’ |
| SARS-CoV-2  nucleocapsid protein gene (N1) | Forward | 5’-GACCCCAAAATCAGCGAAAT-3’ |
|  | Probe | 5’-FAM-ACCCCGCATTACGTTTGGTGGACC-BHQ1-3’ |
|  | Reverse | 5’-TCTGGTTACTGCCAGTTGAATCTG-3’ |

BBQ: blackberry quencher; FAM: 6-carboxyfluorescein; BHQ1: black hole quencher.

Figure S2 – Envelope protein standard curve.

Figure S3 – Nucleocapsid (N1) protein standard curve.
